# Disparities in the Use of Cardiac Rehabilitation after a Myocardial Infarction in the United States

**DOI:** 10.3390/jcm8071006

**Published:** 2019-07-10

**Authors:** Kunal S. Patel, Joshua D. Brown

**Affiliations:** 1 College of Pharmacy, University of Florida, Gainesville, FL 32606, USA; 2 Department of Pharmaceutical Outcomes and Policy, College of Pharmacy, University of Florida, Gainesville, FL 32606, USA; 3 Center for Drug Evaluation and Safety, University of Florida College of Pharmacy, Gainesville, FL 32606, USA

**Keywords:** cardiac rehabilitation, disparities, myocardial infarction

## Abstract

The study’s aim was to identify disparities in the use of cardiac rehabilitation (CR) services. Data were obtained from the 2013 Behavioral Risk Factor Surveillance System (BRFSS) conducted through landline and cellular phones by the Centers for Disease Control and Prevention. Demographic, behavioral, and clinical variables were defined to explore disparities between CR users and non-users. Bivariate chi-square analyses and weighted multivariable logistic regression were used to identify disparities. Analyses were conducted using SAS version 9.4. There were 8506 individuals who had a myocardial infarction (MI) that completed the survey, and 2891 of these individuals reported using CR. The mean weighted CR utilization rate was 31.9% and varied from 17.9% (Hawaii) to 58.9% (Minnesota). Females (adjusted odds ratio (aOR) = 0.73; 0.6–0.88), African Americans (aOR = 0.63; 0.46–0.87), and those in-between the ages of 18 and 49 years-old were less likely to use CR (aOR = 0.54; 0.34–0.86) compared to their counterparts. Individuals who were high school graduates (aOR = 1.57; 1.19–2.07), attended college (aOR = 1.34; 1.01–1.79), or graduated college (aOR = 1.91; 1.41–2.61) were more likely to use CR compared to their counterparts. Non-high school graduates, females, African Americans, and those aged between 18 to 49 should be targeted to increase CR participation.

## 1. Introduction

Each year about 790,000 patients have a myocardial infarction (MI), and of those patients, about 114,000 die from an MI in the United States (US). MIs account for around $11.5 billion in healthcare costs annually. In 2015, there was an estimated annual incidence of about 210,000 recurrent MIs, which accounted for 28.5% of all MIs in the US [1]. Recurrent MI can be overwhelming to patients, due to the high cost of treatment and the increase in mortality and morbidity. After a patient’s initial MI, there is an increased risk of future cardiovascular events, such as recurrent MIs, heart failures, arrhythmias, strokes, and anginas. MI survivors have an increased risk of recurrent MI and a death rate of 5%, which is six times the death rate for patients in the same age group without an MI [2].

When patients have their first MI, most lack the knowledge required to manage their cardiovascular disease and prevent recurrent events. Recurrent MIs and other cardiovascular diseases can be prevented through the use of cardiac rehabilitation (CR). The America Heart Association (AHA) recommends CR as it reduces the risk of future cardiac events by stabilizing or slowing the progress of the current cardiovascular disease, thus reducing the risk of recurrent MI [3]. CR allows providers to assess educational barriers and provide knowledge and resources required for cardiac disease management. CR also focuses on lifestyle changes by providing exercise training, nutritional counseling, and stress management [3]. Patients in an CR program may also undergo aggressive risk factor management, which includes management of hyperlipidemia, hypertension, diabetes mellitus, or smoking cessation.

Studies show that CR is currently underutilized, with use only in 19% to 34% of patients across the US in 2016 [4]. Underutilization may be addressed by targeting specific populations that show low usage of CR programs. Although studies exist on the disparities of CR use, a more thorough investigation is required to gain a better understanding of the factors that are contributing to the low usage of CR. This will allow us to create CR programs that will target different groups of people, increase the use of CR programs, and lead to an overall decrease in mortality and morbidity to those with cardiovascular diseases and an overall decrease in cost related to cardiovascular diseases. This study sought to identify disparities for CR use post-MI in a nationally representative sample using publicly available survey data.

## 2. Methods

Data were obtained from the 2013 Behavioral Risk Factor Surveillance System (BRFSS), which is a project between all 50 states in the US, the District of Columbia, Puerto Rico, Guam, and US Virgin Islands performed by the CDC’s Population Health Surveillance Branch. BRFSS collected data on preventive health practices and behaviors that are linked to chronic diseases, injuries, and preventable infectious diseases. Participants were chosen from a randomly selected list of telephone and cell-phone numbers from a target population of noninstitutionalized US citizens who were 18 years of age or older. Data was then collected and weighted by a statistical method called raking (iterative proportional fitting). Respondents were weighted to be demographically representative of the US population.

We restricted the sample to respondents who reported a previous MI and who resided in a state (n = 16) whose questionnaire included questions about CR. Included states are demographically and geographically varied and are listed in Table 1. Respondents who were 18 years or older were asked, “Following your heart attack, did you go to any kind of outpatient rehabilitation? This is sometimes called rehab.” Demographic, behavioral, and clinical variables were defined to explore disparities between CR users and non-users. Bivariate chi-squared analyses and multivariable logistic regression were used to identify disparities among those who reported use of CR and those that did not. BRFSS survey weights were applied to all analyses to make the results generalizable to the overall population for each state. Analyses were conducted using SAS version 9.4 using an α = 0.05 for statistical significance testing (SAS Institute, Cary, NC, USA).

## 3. Results

In BRFSS and within the 16 states with a CR questionnaire, there were 8506 individuals who had an MI. Of those, 2891 individuals reported “Yes” to using CR. Table 1 reports the individual characteristics such as their age, sex, race, income, and many others. The mean weighted CR utilization rate was 31.9% and varied from 17.9% (Hawaii) to 58.9% (Minnesota). More men (35%) used CR compared to women (26.7%). Caucasians (35%) are more likely to use CR services compared to African Americans (21.6%), Asians (19.3%), Hispanics (22.8%), and other races (25.4%). Individuals who graduated college (44.6%) or high school (33.6%) were more likely to use CR services than those individuals who never graduated high school (21.4%). Individuals who are 80 years or older (40.1%) are more likely to use CR services than those who are in the age groups of 60–79 (36.8%) and 18–59 (20.8%). Individuals who had an income of $75,000 or more (44%) were more likely to use CR services than those who earned less than $75,000 (37.8%), $50,000 (35.6%), and $25,000 (27%).

Table 2 reports the adjusted odds ratio (aOR) and 95% confidence intervals. Females (aOR = 0.73; 0.6–0.88) were less likely to use CR services compared to males. Individuals in-between the ages of 18 and 49 years-old were less likely to use CR (aOR = 0.54; 0.34–0.86) compared to those individuals who are older than 75 years of age. African Americans (aOR = 0.63; 0.46–0.87) were less likely to use CR services compared to Caucasians. Individuals who were high school graduates (aOR = 1.57; 1.19–2.07), attended college (aOR = 1.34; 1.01–1.79), or graduated college (aOR = 1.91; 1.41–2.61) were more likely to use CR compared to those individuals who did not graduate high school. Individuals who exercised during the past 30 days were more likely to use CR (aOR = 1.3; 1.09–1.55) compared to those individuals who had not exercised in the past 30 days during that time. Individuals who were blind or had difficulty seeing (aOR = 0.49; 0.64–0.9) were less likely to use CR services compared to those individuals who were not. Individuals who had difficulty walking (aOR = 0.76; 0.59–0.97) were less likely to use CR compared to those individuals who do not. Individuals who had a prior stroke were more likely to use CR (aOR = 1.33; 1.08–1.65) compared to those individuals who have not had a stroke previously. State, as a stratification variable in the adjusted models, did not change the magnitude or direction of any aOR which showed consistency in disparities between states despite large variation in baseline utilization rates.

## 4. Discussion

Previous studies have shown the many advantages of using CR for cardiovascular diseases, yet CR remains underutilized. Data presented in this research shows that the prevalence of CR use is 31.9%. Low CR attendance can be attributed to disparities that limit access to CR. A recent systematic review and meta-analysis looked at possible disparities in CR participation in the US and found that “older patients, women, patients with less education, unemployed individuals, and uninsured patients are participating less” than their counterparts [5]. Discrepancies between, for example, our findings for age groups compared to these studies are driven by differences in how age is categorized and the base population (e.g., Medicaid insurance). Our study provides a representative population regardless of age, insurance coverage, and other factors and may be considered more broadly for the US population.

Our study shows that females, African Americans, and those in-between the ages of 18 and 49 years-old were less likely to use CR services compared to their older counterparts. Individuals who are blind or have difficulty seeing and those who have difficulty walking were also less likely to use CR services compared to their counterparts. Individuals who did not graduate from high school were less likely to use CR services compared to those who graduated high school and college. Groups should be targeted by increasing awareness of CR programs to increase usage in these groups. With this information, we can create variations of CR programs to help target and increase usage of CR services by these groups. Increasing the use of CR services will be a societal benefit, as it will increase quality-adjusted life-years and reduce the expenditure on cardiovascular diseases. Additional studies are needed to better understand the underlying reasons for disparities and to create CR programs to target those disparities.

The AHA and American College of Cardiology recommend CR (Class 1a Recommendation) for different cardiovascular disorders like MI. CR is recommended to be started within 12 months post-MI, per AHA guidelines [6]. Recommendations are based on the many benefits of CR. CR has been shown to reduce mortality by 20–30% after MI through exercise training, and improved control of cardiovascular risk factors [7]. Studies have also shown a 21% reduction in 5-year mortality for CR patients compared to non-users of CR [8]. CR has also been shown to lower total cholesterol and triglycerides levels, while improving weight control and management of comorbidities [9]. Increasing the use of CR in post-MI patients can lead to decreased hospitalizations, thus decreasing the hospitalization cost. A Cochrane systematic review and meta-analysis of 63 studies with 14,486 participants showed that CR led to a reduction in cardiovascular mortality and risk of hospitalizations [10]. Similar studies have shown reductions in all-cause mortality of 15–28% and reductions in cardiac mortality of 26–31% from using CR services [11].

Studies have shown the many advantages of using CR for cardiovascular diseases, yet CR remains underutilized. Current studies show that participation in CR services ranges from 19% to 34% across states in 2016 due to varying availability of CR services in the United States [4]. Studies have also shown that among Medicare patients with an MI, only 20% of the patients used CR services [12]. Low CR attendance can be attributed to disparities that limit access to CR. Previous studies show that the use of CR services was higher in men than in women. Married MI survivors also have a higher use of CR services compared with unmarried persons. MI survivors with a higher education were more likely to use CR services, and MI survivors with a higher income had a higher prevalence of CR use [13]. Other studies showed that Caucasians are more likely to be referred by a physician for CR services compared to other races [14]. Women with lower incomes were less likely to be both referred and enrolled in CR programs. Of those women, African American women were less likely to be referred and enrolled in CR programs [15].

From previous studies, CR has shown to reduce morbidity and mortality for patients that suffer from different cardiovascular diseases. CR can be a societal benefit, as it will increase quality-adjusted life-years and reduce the expenditure by billions spent on cardiovascular diseases. Recurrent MIs can be greatly reduced by the use of CR services, but only 31.9% of patients used CR services in this study. Increasing the utilization of CR should be a high priority due to the low use of CR. Underutilization of CR services are due to the disparities in different population groups that can be targeted. New programs should be implemented to provide support for those population groups who are underutilizing CR services. Data presented in this research can be used to identify and target those groups. Increasing the use of CR services in these target groups will decrease the morbidity and mortality rate of recurrent MI.

Our study has various limitations. The BRFSS data is self-reported by individuals, which limits the data due to the possibility of recall-bias. This can lead to an underestimation of the number of individuals who had used CR services or those who had a prior MI. The BRFSS data was limited as it only accounted for data from 16 different states and only 8506 individuals. Lastly, due to the limited number of states and individuals who participated, this study may not be reflective of the true demographics that represents the US population. We evaluated this by stratifying the model by state and observed that state variables did not significantly interact with point estimates. Thus, while states have very different baseline rates of CR utilization, the effect of demographic and clinical characteristics may be consistent, and these results are considered generalizable across the US population. More work is needed to distinguish why inter-state disparities exist and specific interventions targeted to increase overall utilization.

## 5. Conclusions

CR services remains underutilized at 31.9% of individuals with recurrent MI, even after the fact that CR services have been shown to decrease mortality and morbidity. Improving awareness of CR services and providing education on CR services to both doctors and patients may increase the usage of CR services.

## Figures and Tables

**Table 1:** Characteristics of post-myocardial infarction (MI) sample population surveyed.

Characteristics	Utilized CR Services (%) ^1^	95% CI
**State ^2^**		
Arizona	23	(22.5–23.5)
Arkansas	29.3	(29–29.6)
District of Columbia	21.3	(21.3–21.4)
Florida	29.6	(28.2–31)
Georgia	28	(27.5–28.6)
Hawaii	17.9	(17.8–18)
Iowa	53.5	(53.2–53.8)
Minnesota	58.9	(58.4–59.5)
Mississippi	27.2	(26.9–27.4)
Missouri	35	(34.4–35.5)
North Dakota	50.9	(50.8–50.6)
Oregon	26.3	(26–26.5)
South Carolina	35.9	(35.5–36.3)
Tennessee	20.8	(20.2–21.4)
Washington	30.7	(30.3–31.0)
Wisconsin	55.7	(55–56.5)
**Sex ^2^**		
Male	35	(33.3–36.8)
Female	26.7	(25.6–27.8)
**Race ^3^**		
Caucasian	35	(32.2–35.9)
Black	21.6	(24.4–25.2)
Asian	19.3	(19.2–19.8)
Hispanic	22.8	(17.1–17.8)
Other	25.4	(22.1–22.5)
**Education ^2^**		
Never Graduated High School	21.4	(20.1–22.7)
High School Graduate	33.6	(32.6–34.7)
Attended College	32.8	(31.7–33.8)
College Graduate	44.6	(43.9–45.4)
Age ^2^		
18–59	20.8	(19.8–21.8)
60–79	36.8	(35.4–38.2)
80 or Older	40.1	(38.9–41.4)
**Income ^2^**		
Less than $25,000	27	(25.5–28.6)
Less than $50,000	35.6	(34.6–36.6)
Less than $75,000	37.8	(37.3–38.4)
$75,000 or More	44	(43.2–44.7)
Total Sample Population	31.9	(30–33.9)

^1.^ Percentages weighted according to state population estimates. ^2.^ *p*-alue < 0.001, chi-square test for difference between users and non-users of cardiac rehabilitation (CR). ^3.^ *P*-alue = 0.0012, chi-square test for difference between users and non-users of CR.

**Table 2:** Odd ratios of possible disparities in individuals verses counterparts that use cardiac rehab services.

Characteristics of Individuals Who Use CR vs Counterparts	Adjusted Odds Ratio (aOR)	Scaled Estimate (95% CI)
**Sex**		
Male	Ref.	Ref.
Female	0.73	(0.6–0.88)
**Age**		
75 or Older	Ref.	Ref.
65–74	0.95	(0.75–1.20)
50–64	0.78	(0.58–1.03)
18–49	0.54	(0.34–0.86)
**Race**		
Caucasian	Ref.	Ref.
African Americans	0.63	(0.46–0.87)
Asian	0.45	(0.14–1.51)
Hispanic	0.89	(0.48–1.65)
Other	0.76	(0.49–1.18)
**Income**		
Less than $25,000	Ref.	Ref.
$25,000–$50,000	1.07	(0.86–1.32)
$50,000–$75,000	1.12	(0.84–1.48)
More than $75,000	1.29	(0.96–1.74)
**Education**		
Non-High School Graduate	Ref.	Ref.
High School Graduate	1.57	(1.19–2.07)
Some College or Technical School	1.34	(1.01–1.79)
College Graduate	1.91	(1.41–2.61)
**Comorbidities**		
Stroke	1.33	(1.08–1.65)
Cancer	0.92	(0.77–1.1)
Arthritis, Rheumatoid arthritis, Gout, Lupus, or Fibromyalgia	0.87	(0.72–1.05)
Kidney Disease	1.16	(0.86–1.57)
Diabetes	1.17	(0.95–1.43)
Depressive Disorders	1.24	(1–1.52)
Asthma	0.89	(0.71–1.11)
COPD	0.94	(0.75–1.18)
**Health Behaviors**		
Exercised during the past 30 days	1.3	(1.09–1.55)
Currently Smoke	0.98	(0.68–1.41)
Consumed Alcohol in past 30 days	1.19	(0.99–1.42)
**Disabilities**		
Requires Walking Cane or Wheel Chair	0.81	(0.64–1.02)
Blind or have difficulty seeing	0.67	(0.49–0.9)
Difficulty Concentrating or Making Decisions	1.07	(0.85–1.35)
Difficulty Walking	0.76	(0.59–0.97)
Physical, Mental, or Emotional Condition	0.97	(0.73–1.30)
**Employment**		
Employed	Ref.	Ref.
Unemployed by Choice	1.08	(0.83–1.4)
Unemployed	0.76	(0.57–1.06)
